# Behavior of Polymer Materials Exposed to Aging in the Swimming Pool: Focus on Properties That Assure Comfort and Durability

**DOI:** 10.3390/polym13152414

**Published:** 2021-07-22

**Authors:** Ivana Salopek Čubrić, Goran Čubrić, Vesna Marija Potočić Matković

**Affiliations:** Faculty of Textile Technology, University of Zagreb, 10000 Zagreb, Croatia; ivana.salopek@ttf.unizg.hr (I.S.Č.); marija.potocic@ttf.unizg.hr (V.M.P.M.)

**Keywords:** aging, polyester, polyamide, elastane, yarn, swimming pool water, tensile properties, microscopy, knitted fabric, drying

## Abstract

The degradation of polyamide (PA) and polyester (PES) polymers is under intense study due to growing concerns about the accumulation of plastics in soils and oceans. Previous studies confirm that ageing degrades PA and PES at the molecular level. However, researchers have not addressed the development of protocols for aging textile materials in swimming pools, and few data are available on the effects of aging on comfort and durability. This research addresses the development of the aging protocol for PA and PES swimwear materials, its implementation, and the evaluation of properties that assure comfort and durability after specific periods of exposure. The tests include microscopic analysis, tensile tests, determination of fluid transport phases and drying period. The results revealed changes in the surface of the material in terms of fibrillation (more pronounced after outdoor aging). There is a positive correlation between the exposure duration and the breaking force (R^2^ ranges from 0.85 to 0.98), with a stronger correlation for the PA materials. The decrease in breaking force due to aging is up to 40%, indicating significantly reduced durability. The change in breaking force follows the changes in mass (R^2^ = 0.867). In terms of comfort assurance, outdoor aging of materials should have a greater negative impact than indoor ageing.

## 1. Introduction

The degradation of PES and PA polymers is being intensively studied, mainly due to growing concerns around the accumulation of plastics and microplastics in soils and oceans, to which textile waste also contributes. Polyamide 6 and polyamide 6.6 are commonly used in all textile products. Polyamide blends with elastane are the most popular fiber blend for swimwear today. Polyester fibers are used for the long lasting shape retention of swimsuits. 

Polyamide 6 and polyamide 6.6 have excellent wear and abrasion resistance to chemicals and oil, and a good balance of mechanical properties. Polyamides tend to absorb moisture, which can affect their properties. Acids can lead to hydrolysis by nucleophilic substitution of the amino link, photolysis leads to scission (breaks in the polymer chains) of the amide linkage [1]. Polyesters are formed by a condensation reaction very similar to the reaction used to produce polyamide. It is commonly used in various textile products. Thermoplastic polyesters have similar properties to PA 6 and PA 6.6, but have lower water absorption and a higher dimensional stability than polyamides. UV radiation absorbed by polyesters leads to scission. In the presence of water, hydrolysis occurs [1]. The influence of UV radiation, water, temperature, humidity and chemical effects has been widely studied. Arhant et al. simulated modification in the physical-mechanical properties of PET polymers made by UV radiation and seawater by aging of PET in water at temperatures ranging from 80 to 110 °C up to 150 days. Embrittlement happens with scission when the polymer molar mass drops below 17 kg/mol during hydrolysis. The brittle state corresponds with a significant decrease in mechanical properties [2]. Chaisupakitsin et al. observed the change in degradation of PET bottle polymers induced by UV radiation and the effect of degradation on drinking water, style and effervescence. After 8 months of sunlight exposure, the tensile strength decreased and the pH of the water changed from acidic to basic for all samples [3]. A new mathematical model for the hydrolytic degradation of PET was proposed by Dubelley et al. The effects of humidity and temperature are included in the model. The model agrees with the data obtained from a literature study [4]. 

Photodegradation of PET fibers under accelerated aging conditions was studied at the nanoscale using resonance-enhanced atomic force microscopy and infrared spectroscopy was investigated by Nguyen-Tri [5]. PET fibers degrade in cementitious alkaline composites, but Rostami et al. concluded that in most applications, the properties of the composite are still improved by PET fibers [6]. An experimental study on the hydrolysis of PA6 in water without oxygen is presented by Deshoulles et al.; 250 microns thick film samples were immersed in oxygen-free water for 2 years. Chain scission, an increase in crystallinity and a sharp increase in water content were observed. A new kinetic model considering water content increase is proposed. The model is used to predict the leakage of macromolecules from the polymer into the oceans [7].

Reactive molecular simulations are used in an Arash study to predict the effect of water on thermal degradation of PA 6.6. The influence of water content on the activation energy and the pre-exponential factor of the cleavage reactions is investigated. A predictive tool to study the long-term thermal degradation of PA 6.6 is offered [8]. Degradation of PA membranes by hydrogen halides and sulfuric acid was investigated by Jun et al. Characterization was carried out by SEM, FTIR and XPS. Sulfuric acid did not cause any changes in the membrane, but hydrogen halides changed the physicochemical properties of the membrane. The results can be used in recycling and industrial processes [9]. 

Thermal oxidation of polyamide 6.6 between 140 °C and 200 °C is investigated in the study by Pliquet et al. The structural changes are monitored by infrared and UV spectroscopy and the microstructure was evaluated by DSC. Vickers µ-hardness was used to evaluate the physical-mechanical properties. Correlations were observed between the changes in chemical structure, change in microstructure and physical-mechanical properties [10]. The degradation of PET and PA in swimsuits is less studied, but Apoloni Cianek simulated the degradation of PET, which is used to manufacture swimsuits in a swimming pool environment. Thermal analysis showed that the degradation occurs in the crystalline phase of the polymer. FTIR analysis showed that the reactions that take place during the degradation process involve the breaking of the polymer chains. Morphological analysis of SEM has shown a decrease in the average diameter of PET samples [11]. In addition to PA and PET, the third polymer fiber used in swimsuits is polyurethane elastane fiber (Spandex), which is used for its stretchability. Marjo et al. studied swimsuits containing an elastane component made from polyetherurethane or polyesterurethane polymers. Hydrolysis was confirmed by thermal testing using ATR-FTIR spectroscopy. The oil on the material was the decomposition product of the polyester component of elastane [12]. Elastane is sensitive to high temperatures. A study by Yin suggests separating PA and elastane in blended fabrics for recycling by heating the material, which damages the elastane but not the PA [13]. All these studies confirm that ageing degrades PA, PES and elastane at the molecular level, but ageing also changes the properties of the textiles. The change in fabric properties further affects the comfort of the material, where the material must provide an optimum or minimum satisfactory level [14], and the durability of the material.

In a study by Salopek Čubrić et al. [15], a significant decrease in all parameters de-scribing tensile properties of PET knitwear was observed after outdoor weathering, but structural parameters after weathering, such as horizontal and vertical density, remained similar. The heat resistance of polyester fabric also remained at almost the same level [14]. In another study, the water vapor resistance of PA- and PET-coated fabrics decreased after outdoor weathering. The average reduction in water vapor resistance after summer weathering was 11.4% and after winter, weathering was 16.7% [16]. The heat resistance of PES-coated PA and PET fabrics decreased by 13% and 25% after 3 months of summer and winter weathering, respectively, in a study by Potočić Matković et al. SEM analysis con-firmed deterioration of the PES layer unrelated to the PA and PET fabric substrate [17]. The deterioration of the PA and PET substrate influenced the decrease in elongation properties, but mainly the decrease in breaking forces, especially after summer exposure [18].

To the best of the authors’ knowledge, researchers have not previously addressed the development of protocols for aging materials in swimming pools that coincide with athlete training and continued use to provide an overview of material performance after specific aging. Therefore, this research aimed to: (i) develop specific aging protocols for swimwear; (ii) implement protocols in the material aging process; (iii) investigate the material properties that are important for durability and comfort in use; and (iv) analyze the effect of aging on material properties. In view of their purpose, the polymer materials were aged in swimming pool water (separately indoor and outdoor), washed in tap water and dried (separately indoor and outdoor). A comparative evaluation was carried out using various tests that are important in the evaluation of aged materials. The tests included microscopic analysis (visual observation—material appearance of non-aged and aged materials, de-termination of horizontal, vertical and overall loop density, measurement of maximum area of voids within loops), tensile tests (breaking force, breaking elongation), duration of fluid transport phases and total drying period of the material.

## 2. Materials and Methods

### 2.1. Material Selection

In this work, the properties of different types of materials and their changes due to aging were studied. For this purpose, a representative set of materials was selected, consisting of two polyester and two polyamide materials (produced by Pletix company, Kamanje, Croatia). All the materials used are weft-knitted materials, the structure of which is built up of basic units called “loops” and dyed blue. During the production of the materials, the elastane component is also added as a plating component. The main parameters of the selected materials in terms of fiber composition are shown in Table 1.

### 2.2. Methods of Measurement

In the experimental part, the following physical-mechanical properties of selected materials are investigated: horizontal, vertical and overall density, the maximum area of voids, tensile properties (breaking force, breaking elongation), duration of fluid transport phases and total drying period.

#### 2.2.1. Microscopy Analysis

The morphology of the materials was visualized using a Dino-Lite Edge AM7915MZT digital microscope (Dino-Lite, Almere, The Netherlands). The microscope is equipped with a 5-megapixel edge sensor, the EDR function (Extended Dynamic Range), which recovers the details of darker or lighter areas within the object by stacking images at different exposure levels, and the EDOF function (Extended Depth of Field), which automatically stacks images at different focus levels to improve the depth of field on rough or uneven surfaces [19].

**Figure 1:**
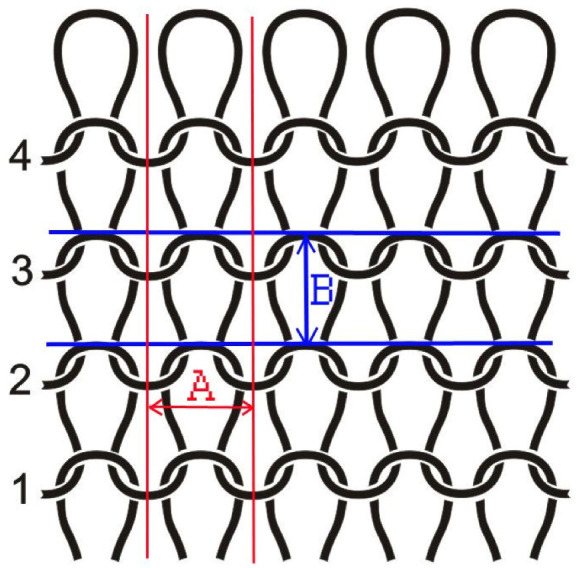
Structure model of knitted fabric indicating segments A and B.

Prior to testing, all samples examined were cut in 100 × 100 mm size under controlled environmental conditions at an air temperature of 20 ± 2 and relative humidity of 60 ± 5 °C. A magnification of 200× was used for microscopic analysis. Image analysis was performed using DinoCapture 2.0 professional software (Dino-Lite, Almere, The Netherlands). The analysis focused on determining the horizontal density (Dh), vertical density (Dv) and maximum area of voids. Dh and Dv are determined by measuring segments A and B (shown in the structure model, Figure 1).

The length of segments A and B is further used in Equations (1) and (2) to determine Dh and Dv, respectively. For each specimen, 10 replicates were performed. Finally, the values of Dh and Dv are used to calculate the overall loop density (D) according to Equation (3).

Dh = M_u_/A (1)

Dv = M_u_/B (2)

D = Dh × Dv(3)

where: Dh (cm^−1^)—horizontal loop density, M_u_—unit of measurement (1 cm), Dv (cm^−1^)—vertical loop density, D (cm^−2^)—overall loop density.

The maximum area of voids within loops is determined from the microscopic image using the “Polygon” tool. For the determination of the surface of voids, 10 microscopic images were taken of each sample.

#### 2.2.2. Testing the Mass per Unit Area

According to the standard ISO 3801 [20], the mass per unit area represents the mass of one square meter of a flat product in grams. The test of mass per unit area is carried out using an analytical scale with an accuracy of 0.0001 g. Round samples of the size 1 dm^2^ are prepared for the measurement.

#### 2.2.3. Tensile Testing

The tensile properties of the knitted fabric was tested according to the procedure de-scribed in the international standard ISO 13934-1 [21]. Statimat M (Textechno, Mönchengladbach, Germany) tensile tester from was used for the measurement. The accuracy of the tensile tester corresponds to class 1, as defined in the standard EN 10002-2 [22]. In this context, the error of the recorded maximum force at any point of the fabric test is ±1%, while the error of the jaw distance is ±1 mm. The tensile tester is equipped with two clamps, one of which is stationary and the other moves at a constant speed to the point of fabric breakage. The device and the whole system are free from deflection. The gauge length has been set to 100 mm. The strip test method is used for the measurement, which involves the use of strip specimens with dimensions 50 × 200 mm. The atmosphere for preconditioning, conditioning and testing was the same as the standard testing atmosphere, i.e., temperature 20 ± 2 °C and relative humidity 65 ± 3%. The specimens were conditioned for 24 h in standard atmosphere and relaxed condition. The following segments describing tensile properties were measured and further observed:

- Force at rupture, i.e., the breaking force—a force recorded by the tester at the point of the specimen rupture during the test;
- Elongation at rupture, i.e., the breaking elongation—measured elongation of the tested specimen corresponding to the force at break.

**Figure 2:**
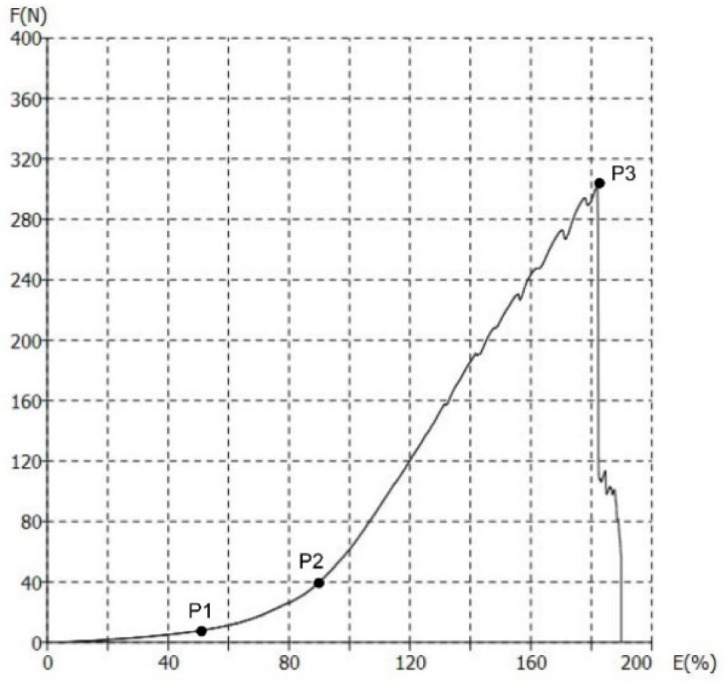
Identification of breakpoints in the regions of elastic, elastoplastic and plastic.

The force-elongation (F/E) diagram of knitted fabric can be divided into three regions, Figure 2. The first region (from 0 to P1) is the elastic region. This region is linear. The structure of the materials has elastic response and the yarn that builds the structure moves within the structure. The second region (from P1 to P2) is nonlinear. In this region, the elastoplastic deformation of the materials occurs, which is caused by the increase of the force. Finally, the third region (from P2 to P3) is again linear. In this region, the deformation of the yarn occurs, resulting in plastic deformation. This deformation is permanent and the point P3 indicates the maximum breaking force, which leads to the complete breakage of the material.

#### 2.2.4. Fluid Transport Phases and Total Drying Period

**Figure 3:**
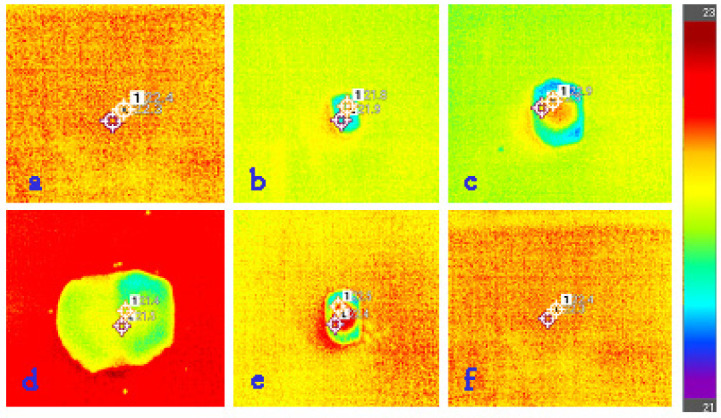
Thermographic images taken in different fluid transport phases: (**a**) material was placed; (**b**) distilled water was dispensed; (**c**) the size of the wetted zone increased; (**d**) the size of the wetted zone increased to its maximum; (**e**) the wetted area shrank; (**f**) the end of the active drying phase.

In order to observe the fluid transport through the material and its drying ability, an E6 infrared camera (Flir Systems Inc., Wilsonville, OR, USA) was used. The camera used has a thermal sensitivity of 0.06 °C and a reading accuracy of ±2%. Before the measurement, the sample was cut into squares of 200 × 200 mm and left horizontally oriented in an indoor environment with an air temperature of 20 ± 2 °C and 65 ± 3% relative humidity for 24 h. Distilled water (0.1 mL) was dispensed with the pipette positioned vertically at a distance of 20 mm from the sample surface. A thermal imaging camera was used to measure the temperature of the sample before the water was deposited. After deposition, the surface temperature changed in the zone of the wetted area, which had a lower temperature than the rest of the sample surface. This change can be easily seen on the display of the thermal imaging camera as the color changes in this zone (Figure 3). 

As the water diffuses over the sample, the size of the wetted zone increases and the temperature of the zones decreases (Figure 3b–d). This phase is called the wetting phase (WP). After that, the water stops expanding, which is associated with the next phase, the static phase (SP). During this phase, there was no significant change in the surface of the wetted area. Finally, when the water began to evaporate, the wetted area shrank (Figure 3e) and the temperature of the area began to return to the average temperature of the non-wetted area. This was the beginning of the active drying phase (ADP). The moment when the temperature of the primary wetted zone reached the temperature of the non-wetted zone was defined as the end of the active drying phase. The static phase (SP) and the active drying phase (ADP) together define the total drying period of the material (TDP). In this experiment, five measurements for each material are used to characterize the observed phenomena. The data were processed in the FLIR Tools^©^ professional thermal analysis software.

#### 2.2.5. Fourier Transform Infrared (FT-IR) Spectroscopy

Samples were analyzed using Fourier Transform Infrared (FT-IR) spectroscopy with Spectrum 100 software (PerkinElmer Inc., Waltham, MA, USA). Four scans were performed for each sample with a resolution of 4 cm^−1^ between 4000 cm^−1^ and 380 cm^−1^.

### 2.3. Aging Protocol

Representative materials (all knitted fabrics) were selected for the experiment described in this paper, as previously described. The material aging protocol included the following steps:

- Selecting the target group;
- Defining the specific training conditions;
- Determining the aging factors;
- Determining the order in which the aging was performed.

In accordance with the main purpose of the selected materials are delimited as a target group recreational swimmers and swimmers at the beginning of a sports career. Their training ritual includes training in the pool water with a duration of 2–6 h per week, giving an average of 3.5 h per week [23]. For this study, the use of materials is simulated for:

- Six weeks (6w), which equates to a total of 21 h of use;
- Twelve weeks (12w), which equates to a total of 42 h of use;
- Eighteen weeks (18w), which corresponds to a total of 63 h of use.

The majority of professional swimmers train in indoor pools, but during the hot season both recreational and professional swimmers train very often in outdoor pools. Training in outdoor pools includes the influence of the sun as an additional aging factor. Therefore, the aging of materials under indoor and outdoor pool conditions is performed separately. The aging was performed under real conditions, during the hot and dry season at the coordinates 45° N, 13° E. The average air temperature was 23 ± 1 °C, the wind speed was 4 ± 1 m/s. The UV indeks (UVI), as a measure of the level of UV radiation, was 7. According to the World Health Organization, UVI 7 is considered as high (the scale is 1–11). Furthermore, there was no precipitation. The concentration of chlorine in the pool water was 0.5 mg/L, and pH was 7.2. Table 2 provides an overview of the aging protocols with the corresponding designation of each protocol.

The order of aging was as follows:

- Immersion of materials in chlorinated swimming pool water for defined periods of time (either indoors or outdoors);
- Rinsing the materials with clean tap water (temperature 30 °C);
- Drying of the materials in the flat state, under natural air circulation (only for the simulation of aging in the indoor swimming pool);
- Drying outdoors in a flat state, under direct sunlight, with an average air circulation of 4 m/s (only for simulating aging in the outdoor pool).

All aged and non-aged materials were tested using the methods as described in Section 2.2.

## 3. Results and Discussion

### 3.1. Results of the Microscopic Analysis

The representative microscopic images of the studied materials taken before aging (Wa) and after the simulated 18-week aging (both outdoor and indoor—Po-18w and Pi-18w) are shown in Table 3. The images have been converted to grayscale to facilitate comparison. As can be seen, the structure of non-aged materials is very uniform, with no signs of deterioration of any kind. The structure of sample KF1 is slightly less visible as this material has been dyed a darker color, which has affected the visibility of the image to some extent, but even then the structure is fairly uniform. As can be seen further, there are noticeable changes in the appearance of the material after aging, i.e., there are changes in the surface of the material in terms of fibrillation. The fibrillation is present on the entire surface of aged materials. The longest protruding fiber has a length of 1.25 mm. Among the presented microscopic images, this phenomenon is best seen in the materials assigned as KF2 and KF3. Additional analysis of a large set of microscopic images taken on different segments of the aged materials revealed that the surface deterioration is more pronounced after outdoor aging, i.e., after additional exposure to direct sunlight. This result is in agreement with the results attributed to photodegradation by Nguyen-Tri [5] and chemicals used for disinfecting by Cianek [11], which also showed surface damage to PET fibers. The observed fibrillation on the material surface is expected to have a negative impact on the tactile comfort of the material and to facilitate the pilling. As shown in previous research, textile products, such as swimwear, appeal to the user’s sense and sense of touch [24], which in this case is negatively affected by aging in KF2 and KF3 materials. At this point, there was no visually observed deterioration of the material structure that might indicate a significant change in other properties. There was no fading of the dyestuff on aged samples.

Polymeric materials produced with knitting technology have the most unstable structure compared to materials produced with other technologies. The data on fabric density and its changes due to exposure to various influences are quite valuable in knitted fabrics. They give some insight into the structural transformation that can affect other material properties. The changes in material densities (expressed as percentages) compared to the densities of non-aged materials are shown in Table 4. The values shown include the average density of loops in the horizontal and vertical directions, as well as the overall loop density. To illustrate the changes in material densities (in the horizontal and vertical direction and in both directions), the results are presented for each material and after the longest exposure time. Reduced knit density, in either direction, compromises comfort or material aesthetics. It can be assumed that it is more associated with a loss of elastic properties in PET than in PA blends.

Another material parameter determined by microscopic analysis was the maximum area of voids within the material structural elements. Despite the fact that this analysis is usually performed using only magnifying glass, microscopic analysis was used for this research because it provides more reliable and precise data with high reading accuracy. The results in Table 5 show that the effect of aging factors on the materials causes a significant increase in the maximum area of voids on the polyamide materials. Namely, the maximum area of voids for polyamide materials increases from 0.003/0.005 mm^2^ (non-aged specimen) to 0.007 mm^2^. There is an obvious change in the ideal shape of the basic unit of the structure, which may affect the change in other properties of the material. It is important to note that no such significant change was observed in polyester materials (the maximum area of voids is 0.001 and 0.002 mm^2^ before aging, and 0.001–0.003 mm^2^ after aging). Since the values of maximum void area after outdoor and indoor exposure are similar, it is not possible to draw a conclusion with certainty about the influence of individual aging on the observed changes.

### 3.2. Results of the Test of Mass per Unit Area

**Figure 4:**
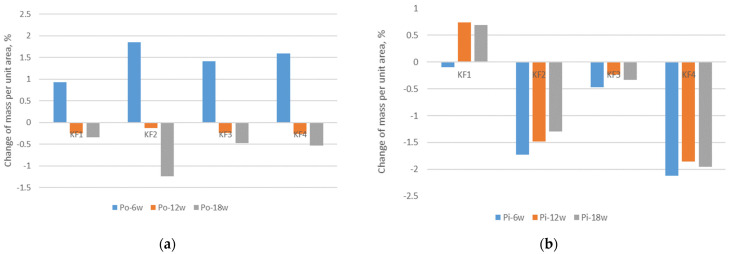
Change in mass per unit area after exposure compared to mass per unit area of non-aged materials: (**a**) outdoor; (**b**) indoor.

In the previous study [6], related to the exposure of polyester material in alkaline media, the positive effect on the reduction of mass per unit area was reported. In contrast to this study, the results presented in Figure 4 indicate a specific behavior of porous knitted structures, especially after outdoor exposure. Namely, for all the studied materials, the mass per unit area increases in the first phase of exposure to outdoor conditions (i.e., after 6 weeks of exposure), Figure 4. This fact should be explained by a rapid and significant change in the fabric structure, leading to a shrinkage of the material, which consequently affects the increase in mass per unit area. With further exposure of the materials, the mass per unit area decreases, indicating that the material structure changes and the material relaxes (i.e., shrinkage decreases). For materials aged indoors, different material behavior is observed in terms of changes in mass per unit area. In particular, the mass of materials with elastane (materials KF2, KF3 and KF4) decreases after exposure. As can be seen, this decrease is not proportional to the duration of exposure. In contrast, the mass per unit area of the polyester material without elastane content increases due to the exposure, which leads to the conclusion that the material without elastane content shrinks more than the material with elastane content. It is to be assumed that fabric KF1, with longer exposure to indoor aging, would follow the behavior of other fabrics.

### 3.3. Results of Tensile Testing

**Figure 5:**
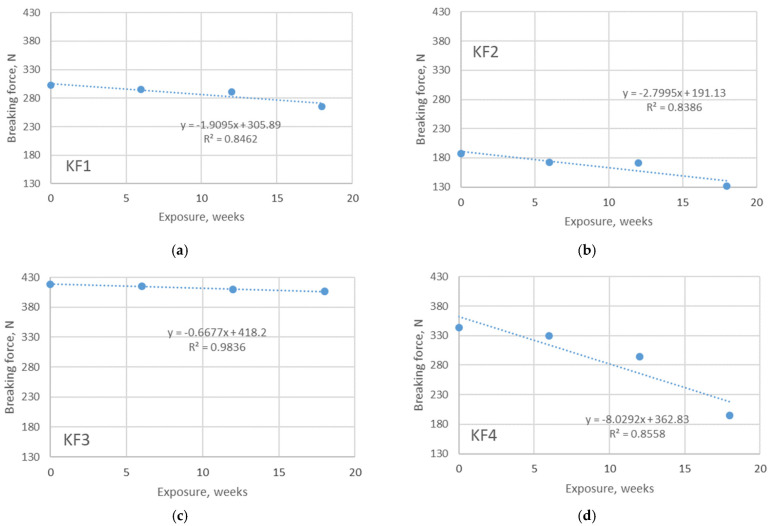
Breaking force of materials exposed to aging in pool water, outdoors. The overview is given for: (**a**) material KF1; (**b**) material KF2; (**c**) material KF3; (**d**) material KF4.

**Figure 6:**
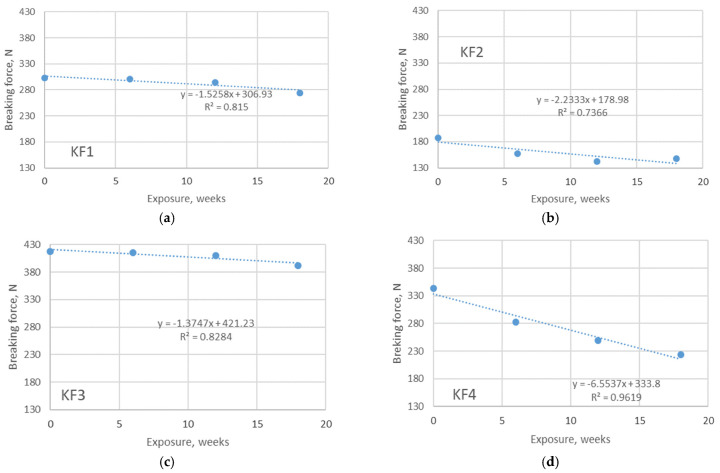
Breaking force of materials exposed to aging in pool water, indoors. The overview is given for: (**a**) material KF1; (**b**) material KF2; (**c**) material KF3; (**d**) material KF4.

The instability of the knitted structure allows the knitted material to fit the body better and is therefore preferable for use in sportswear. The average stresses acting in different types of knitted material for sportswear are rather low. The peculiarity of swimwear for athletes (especially professional athletes) is that such materials fit very tightly to the body to allow faster body movements in the water. At this point, the stresses acting on such a material are much higher than the stresses acting on materials for other purposes. Since the aging process affects the changes in tensile properties, the data on the breaking force are essential to describe issues related to the durability of the material. A significant decrease in the physical-mechanical properties of PET fibers has been previously reported after exposure to UV radiation [3], UV radiation and seawater [2], and on PET and PA fibers after outdoor exposure [14,17]. The results of the tensile tests, which focus on the measured values of the breaking force, are shown in Figure 5 and Figure 6. 

As can be seen from the figures, the values of the measured breaking forces of the non-aged materials are in the range of 187–417 N, being higher for the polyamide materials. After the longest exposure period (18 weeks in this case), the values are much lower and are in the range of 132–265 N. A positive correlation is observed between the exposure duration (as independent variable) and the breaking force (as dependent variable) for both aging in outdoor and indoor environments. The corresponding linear regression coefficients (R^2^) show a strong correlation between the observed variables for all materials aged outdoors (R^2^ ranges from 0.84–0.98). A slightly stronger correlation is present for the polyamide materials (materials KF3 and KF4). The decrease in breaking force due to aging is up to 40% (for material KF4), which indicates that the durability of this material is significantly reduced and its further use for athletes cannot be recommended. The reason for such a result is the fact that the structure of the material, as well as the strength of polyamide and polyester yarns, is strongly negatively affected by immersion in swimming pool water and exposure to sunlight. Compared to the results of aging under indoor conditions, the trend previously observed is the same, but the decrease in breaking force is less pronounced. In this type of aging, the values of breaking force after the longest period of exposure are in the range of 148–391 N. This observation can be explained by the absence of the additional influence of sunlight. The values of R^2^ are in the range of 0.74–0.96, indicating a moderate to strong positive correlation between breaking force and exposure time.

**Figure 7:**
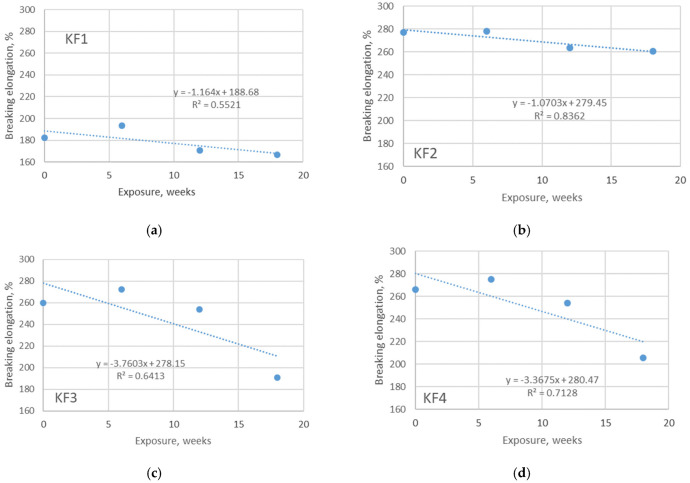
Breaking elongation of materials exposed to aging in pool water, outdoor. The overview is given for: (**a**) material KF1; (**b**) material KF2; (**c**) material KF3; (**d**) material KF4.

**Figure 8:**
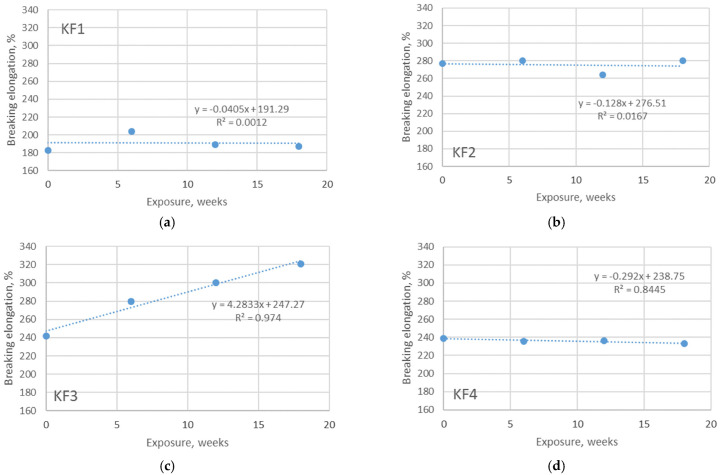
Breaking elongation of materials exposed to aging in pool water, indoors. The overview is given for: (**a**) material KF1; (**b**) material KF2; (**c**) material KF3; (**d**) material KF4.

Materials made using knitting technology are known to have improved comfort properties, especially when elastane yarns are also used for plating. The previously published researches showed that the content of elastane in the material structure has a significant influence on the elastic properties (primary elastic recovery) of the materials [25,26]. The results of the measured breaking elongation of the investigated materials confirmed this evidence. More precisely, the breaking elongation of non-aged polyester material without elastane component is only 182 N, while the breaking elongation of the polyester material with the addition of 15% of elastane component increases by 52% (Figure 7). The effect of smaller differences in elastane content (in this case 20% and 22%, respectively, as defined in Table 1) has no effect on the changes in breaking elongation that can be considered significant. The experiment presented in this study provided insight into the changes of breaking forces due to aging. Unlike the breaking force, the breaking elongation does not decrease with the same trend over the exposure time. The graphs presented in Figure 7 show a decrease of breaking elongation with increasing exposure duration. The coefficients describing this relationship range from 0.55 to 0.87, mostly indicating a medium correlation. The graphs shown in Figure 8 indicate that there is no uniform change in breaking elongation when the materials are aged indoors. This primarily relates to polyester materials where the relationship between the observed variables (breaking elongation and exposure duration) is very weak (R^2^ is 0.0012 and 0.0167 respectively). These results led to the conclusion that the chemicals and pool water do not affect the changes in the breaking elongation of the materials in the same way that exposure to sunlight does. When the measured values of breaking elongation are placed directly in the context of the measured mass per unit area (through correlation coefficients shown in Table 6), it can be seen that the change in breaking elongation strongly follows the changes in mass per unit area (the correlation coefficient is 0.867). In terms of comfort assurance, the results showed that outdoor aging of materials should have a stronger negative effect than aging indoor. 

**Figure 9:**
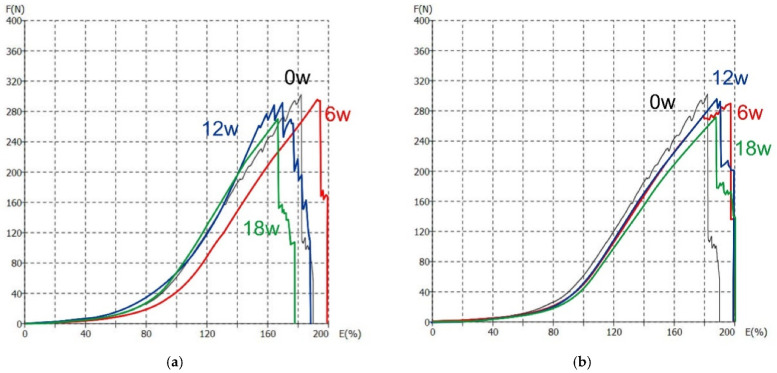
The F/E curves for material KF1: (**a**) In outdoor conditions; (**b**) in indoor conditions.

The F/E curves for material KF1 measured under outdoor and indoor conditions are shown in Figure 9. To illustrate the behavior of the material in the elastic, elastoplastic, and plastic regions, F/E curves are further extracted for each material and compared for non-aged materials and materials that have been subjected to aging (6w, 12w, and 18w). For illustration, the F/E curves for material KF1 are shown in Figure 9 (for both outdoor and indoor measurement conditions). As can be seen from Figure 9a, the first region, i.e., the elastic region, is linear and corresponds to an estimated elongation of 58% (point P1). This region is very important for the functionality and durability of materials and is therefore used in the process of material design. The second region starts at elongation of 58% (point P1) and ends at 90% (point P2). This is an elastoplastic region, which is considered to be a boundary region between the elastic and plastic. The stretching of the materials of material KF1 with the tensile tester was carried out up to the breaking point, which in this case corresponds to the force of 302 N and the elongation of 182% (point P3).

**Figure 10:**
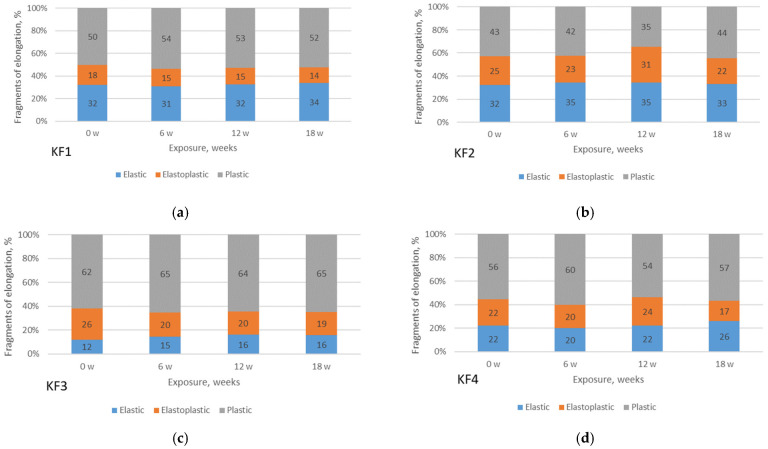
Fragments of elongation of materials exposed to aging outdoors: (**a**) material KF1; (**b**) material KF2; (**c**) material KF3; (**d**) material KF4.

**Figure 11:**
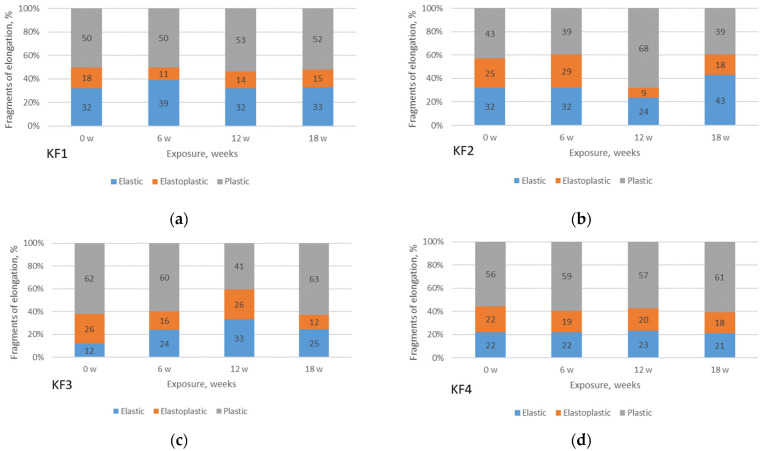
Fragments of elongation of materials exposed to aging indoor: (**a**) material KF1; (**b**) material KF2; (**c**) material KF3; (**d**) material KF4.

The F/E curves of each material and each condition are further used to define the points associated with the regions and to define the fragments of elongation of the materials (expressed as percentages), Figure 10 and Figure 11.

From Figure 10, it can be seen that the material KF3 has the lowest fragment of elastic region for all exposure duration (12% for non-aged; 15% for 6 weeks of exposure; 16% for 12 weeks; 16% for 18 weeks). On the contrary, material KF2 has the highest fragment of elastic region (32%; 35%; 35%; 33%, respectively). It is also observed that PA materials (KF3 and KF4) have lower fragments of the elastic region than PES materials (KF1 and KF2). It must be emphasized that for all materials the fragment of the elastic region increases with the aging of the material. For all the materials studied, it should be noted that the elastoplastic region is between 14% and 31%, while the plastic region is between 35% and 65%. Material KF3 has the higher plastic region range (62–65%).

Looking at Figure 11, where the material is aged indoors, again the KF3 material has the lowest fragment of the elastic region (12% for non-aged; 24% at 6 weeks exposure; 33% at 12 weeks; 25% at 18 weeks). However, the KF1 material has the lowest fragment of elastic region in indoor aging (32–39%). No regularities are observed in the change of fragment of the elastic region changes due to aging time. For all the materials studied, it should be noted that the elastoplastic region is between 9% and 29%, while the plastic region is between 39% and 68%.

Comparing the relationship between outdoor and indoor aging, no significant differences are observed between the regions for materials KF1 and KF4. For the KF2 material, deviations can be seen at 12 and 18 weeks of exposure, while for the KF3 material this difference already exists at 6 weeks of aging exposure.

### 3.4. Results of Total Drying Period and Fluid Transport Phases

**Figure 12:**
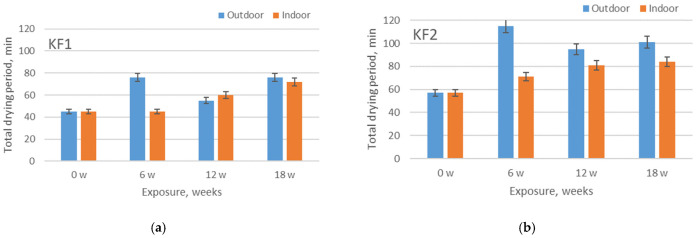

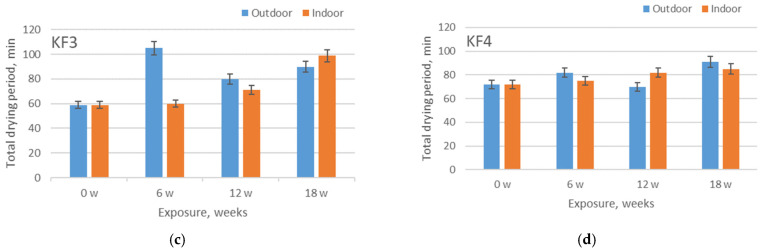
Comparison of total drying rate of materials exposed to aging outdoors and indoors: (**a**) material KF1; (**b**) material KF2; (**c**) material KF3; (**d**) material KF4.

The determination of drying period of swimwear materials is extremely important for achieving comfort during the phases when the athlete is not in the water but waiting next to the pool to continue swimming (e.g., in competitions). The polyester and polyamide materials are therefore advantageous for this use due to their short drying time (compared to other textile fibers). The comparison of total drying rate of materials exposed to aging under outdoor and indoor conditions in relation to the wetted area of the material, is shown in Figure 12. The results confirm the influence of exposure to sunlight on the prolongation of drying period. More specifically, in 9 out of 12 cases, the drying period is longer for the materials aged outdoors. This prolongation is up to 64%. The changes in drying period cannot be explained by the changes in mass per unit area, since the correlation of these parameters is rather weak (−0.150). Therefore, these changes should be discussed in the context of the combined structural and surface changes, i.e., the described process of fibrillation (as explained in Section 3.1) and the changes in the size of voids within the elementary units. Regarding the influence of the polymer type and the addition of the elastane content, the results confirmed that these two parameters do not have a significant influence on the total drying period.

**Figure 13:**
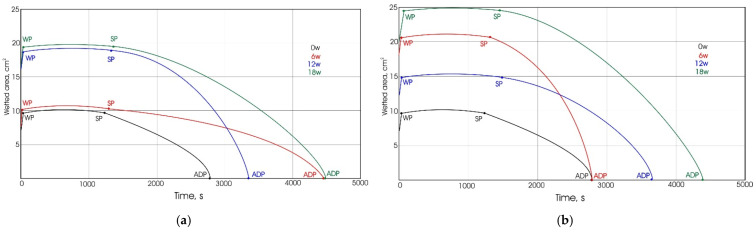
Identification of breakpoints marking the end of the fluid transport phases (WP, SP and ADP): (**a**) outdoor; (**b**) indoor.

To further explain the fluid transfer, Figure 13 shows the identification of breakpoints marking the end of the fluid transfer phases (WP—wetting phase, SP—static phase, and ADP—active drying phase) for material KF1. This identification revealed an interesting behavior of knitted material. For both observed aging conditions, the WP phase duration is very short (up to 30 s) in all cases (0w, 6w, 12w and 18w), which is consistent with the properties of polymer materials. As aging increases, the duration of SP increases (for both outdoor and indoor aging). Finally, the duration of the active drying phase increases by increasing the exposure time. It can be seen that there is some uniformity of increase for the indoor aged materials, whereas there is no uniformity for the outdoor aged materials (as seen for the duration of ADP phase of materials aged for 6 and 12 weeks).

**Figure 14:**
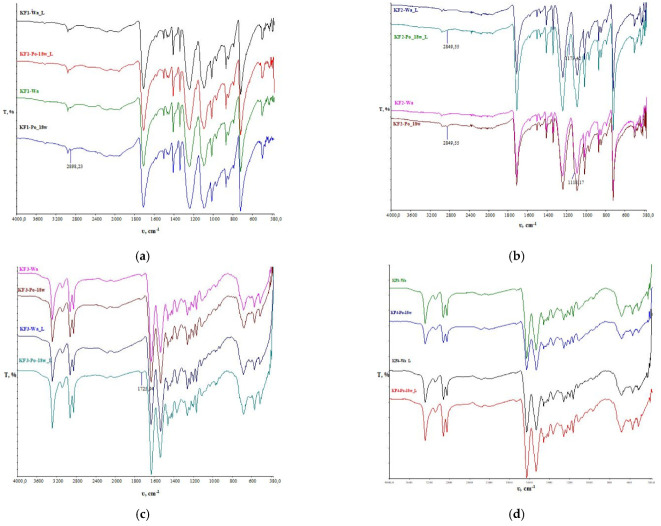
Fourier Transform Infrared (FT-IR) spectroscopy spectrum of the samples before aging (Wa) and after 3 weeks of outdoor aging (Po-18w) for the face (L) and back side of the material: (**a**) KF1; (**b**) KF2; (**c**) KF3; (**d**) KF4.

From the obtained spectral bands of the polyester material (sample KF1, Figure 14), no major physicochemical changes were observed in the samples subjected to aging. Minor changes occurred on the reverse side of sample KF1-Po-18w. A more pronounced peak compared to the pre-aged sample is visible at 2898 cm^−1^. The peak intensity at 1171 cm^−1^ is reduced, indicating structural changes due to hydrolysis and photo oxidation within the PES polymer chain. This is also seen for sample KF2.

On the spectral bands of polyamide sample KF3 before and after aging, the presence of peaks on the back of the material caused by vibrations within the carbonyl groups is visible. On the face of sample KF3-Wa subjected to analysis, it is visible that there is no peak in this wave number, but after aging, the peak appears. This indicates the physicochemical changes in the face of the material, which is partially worn off and the response of the back of the material predominates.

Compared to the initial sample, no physicochemical changes were observed in sample KF4 subjected to aging (both face and the back of the sample).

## 4. Conclusions

The results obtained in this research allowed us to reach the following conclusions related to the aging of PA and PES materials in different environments:

- there are noticeable changes in the surface of the material in terms of fibrillation. The surface deterioration is more pronounced after outdoor than indoor aging, which confirms the effect of UV radiation on fibrillation;
- a significant increase in the maximum void area is observed only in the polyamide materials (maximum void area increases from 0.003 to 0.007 mm^2^);
- the mass per unit area increases in the first phase of outdoor aging. With further exposure of the materials it decreases, indicating that the material structure changes—the material relaxes and shrinkage decreases, which is in accordance with the measurement of reduced knit density;
- a positive correlation is observed between exposure duration and breaking force for both outdoor and indoor aging (R^2^ ranges from 0.84 to 0.98), with a slightly stronger correlation for the PA materials. The decrease in breaking force due to aging is up to 40% (for PA), indicating that the durability of this material is significantly reduced. The decrease in breaking force is less pronounced for materials aged indoors;
- the breaking elongation does not decrease with the same trend over the exposure time (R^2^ is 0.55 to 0.87). The change in breaking force strongly follows the changes in mass per unit area (the correlation coefficient is 0.87). In terms of comfort assurance, the outdoor aging of materials should have a greater negative effect than aging indoors;
- determining the drying period of swimwear materials is extremely important for achieving comfort. The results confirm the influence of exposure to sunlight on the increase of the drying period up to 64%;
- the FT-IR analysis showed the physicochemical changes in the surface (face side) of the PA material with elastane (78/22%) only after outdoor aging in the period of three weeks.

All measured properties, with the exception of indoor elongation, confirm the influence of water and chlorine, and especially solar radiation on the polymer degradation and degradation properties of the fabric, as well as a negative influence on wearing comfort. The elongation of the fabric seems to be influenced by complex interactions, which should still be investigated in detail.

## Figures and Tables

**Table 1:** Parameters of a set of materials.

Material Designation	Fiber Composition	
	Ground Yarn, Share	Plating Yarn, Share
KF1	Polyester, 100%	Elastane, 0%
KF2	Polyester, 85%	Elastane, 15%
KF3	Polyamide, 78%	Elastane, 22%
KF4	Polyamide, 80%	Elastane, 20%

**Table 2:** The aging protocols.

Designation	Aging Description	Simulated Aging Duration
Wa	Without aging	-
Po-6w	Aging in pool water, outdoor	Six weeks (21 h)
Po-12w	Aging in pool water, outdoor	Twelve weeks (42 h)
Po-18w	Aging in pool water, outdoor	Eighteen weeks (63 h)
Pi-6w	Aging in pool water, indoor	Six weeks (21 h)
Pi-12w	Aging in pool water, indoor	Twelve weeks (42 h)
Pi-18w	Aging in pool water, indoor	Eighteen weeks (63 h)

**Table 3:** Microscopic images of the materials before aging (wa) and after three weeks of aging outdoor (Po-18w) and indoor (Pi-18w).

	Wa	Po-18w	Pi-18w
KF1	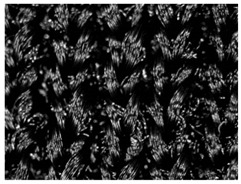	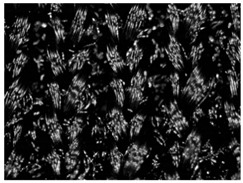	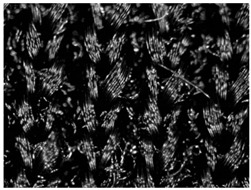
KF2	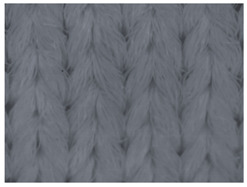	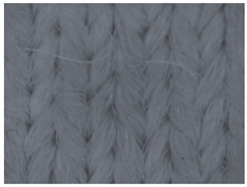	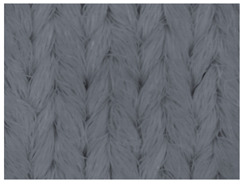
KF3	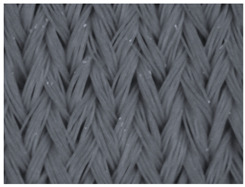	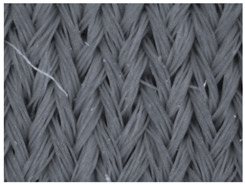	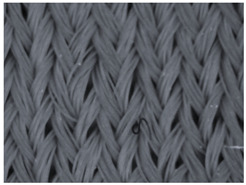
KF4	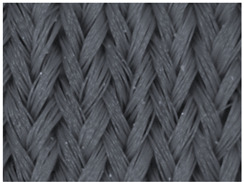	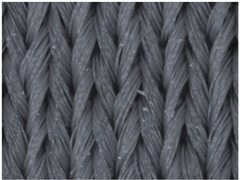	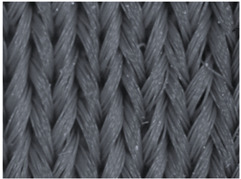

**Table 4:** The changes of densities, expressed in %, in comparison to non-aged materials.

Material	D_h_		D_v_		D	
	Po-18w	Pi-18w	Po-18w	Pi-18w	Po-18w	Pi-18w
KF1	−1.70	−2.22	−1.70	−2.74	−3.44	−5.02
KF2	−4.94	−7.41	−3.52	−3.11	−8.64	−10.75
KF3	−0.09	−0.64	−0.20	−3.54	−0.29	−4.21
KF4	−4.28	−0.41	−0.35	−0.84	−4.65	−1.26

**Table 5:** The maximum area of voids.

Material	Maximum Area of Voids within Loop, mm^2^		
	Wa	Po-18w	Pi-18w
KF1	0.001	0.002	0.001
KF2	0.002	0.003	0.002
KF3	0.005	0.007	0.007
KF4	0.003	0.006	0.007

**Table 6:** The correlation coefficients—mass per unit area, breaking force and breaking elongation.

Variable	Mass per Unit Area	Breaking Force	Breaking Elongation
Mass per unit area	1000		
Breaking force	−0.266	1000	
Breaking elongation	**0.867**	−0.226	1000

Marked correlation is significant at *p* < 0.050.

## Data Availability

Data available in a publicly accessible repository.
